# British Society for Echocardiography and British Cardio-Oncology Society guideline for transthoracic echocardiographic assessment of adult cancer patients receiving anthracyclines and/or trastuzumab: Guidelines and Recommendations

**DOI:** 10.1530/ERP-21-0001

**Published:** 2021-03-03

**Authors:** Rebecca Dobson, Arjun K. Ghosh, Bonnie Ky, Tom Marwick, Martin Stout, Allan Harkness, Rick Steeds, Shaun Robinson, David Oxborough, David Adlam, Susannah Stanway, Bushra Rana, Thomas Ingram, Liam Ring, Stuart Rosen, Chris Plummer, Charlotte Manisty, Mark Harbinson, Vishal Sharma, Keith Pearce, Alexander R. Lyon, Daniel X. Augustine

**Affiliations:** 12 Cardio-Oncology Service, Liverpool Heart and Chest NHS Foundation Trust, Liverpool, UK; 22 Cardio-Oncology Service, Barts Heart Centre, Barts Health NHS Trust, London, UK; 32 Cardio-Oncology Service, Hatter Cardiovascular Research Institute, University College London and University College London Hospitals NHS Foundation Trust, London, UK; 42 Perelman School of Medicine at the University of Pennsylvania, Philadelphia, Pennsylvania USA; 52 Baker Heart and Diabetes Institute, Melbourne, Australia; 62 University Hospital South Manchester NHS Foundation Trust, Manchester, UK; 72 East Suffolk and North Essex NHS Foundation Trust, Colchester, UK; 82 University Hospitals Birmingham NHS Foundation Trust, Birmingham, UK; 92 North West Anglia Foundation Trust, UK; 102 Liverpool John Moores University, Liverpool, UK; 112 University Hospitals of Leicester NHS Trust, Leicester, UK; 122 Royal Marsden NHS Foundation Trust and Institute of Cancer Research, London, UK; 132 Imperial College Healthcare NHS Trust, London, UK; 142 The Shrewsbury and Telford Hospital NHS Trust, Shrewsbury, UK; 152 West Suffolk NHS Foundation Trust, Bury St Edmunds, UK; 162 Royal Brompton and Harefield NHS Foundation Trust and Imperial College London, London, UK; 172 The Newcastle upon Tyne Hospitals NHS Foundation Trust, Newcastle, UK; 182 Belfast Health and Social Care Trust, Belfast, UK; 192 Royal Liverpool and Broadgreen University Hospitals NHS Trust, Liverpool, UK; 202 Department of Cardiology, Royal United Hospitals Bath NHS Foundation Trust, Bath, UK; 212 Department for Health, University of Bath, Bath, UK

**Keywords:** anthracycline, echocardiography, guidelines, HER2 therapy, imaging

## Abstract

The subspecialty of cardio-oncology aims to reduce cardiovascular morbidity and mortality in patients with cancer or following cancer treatment. Cancer therapy can lead to a variety of cardiovascular complications, including left ventricular systolic dysfunction, pericardial disease, and valvular heart disease. Echocardiography is a key diagnostic imaging tool in the diagnosis and surveillance for many of these complications. The baseline assessment and subsequent surveillance of patients undergoing treatment with anthracyclines and/or human epidermal growth factor (EGF) receptor (HER) 2-positive targeted treatment (e.g. trastuzumab and pertuzumab) form a significant proportion of cardio-oncology patients undergoing echocardiography. This guideline from the British Society of Echocardiography and British Cardio-Oncology Society outlines a protocol for baseline and surveillance echocardiography of patients undergoing treatment with anthracyclines and/or trastuzumab. The methodology for acquisition of images and the advantages and disadvantages of techniques are discussed. Echocardiographic definitions for considering cancer therapeutics-related cardiac dysfunction are also presented.

Anju Nohria, MD, served as the Editor-in-Chief for this paper. Juan Carlos Plana Gomez, MD, served as the Guest Associate Editor for this paper. The authors attest they are in compliance with human studies committees and animal welfare regulations of the authors' institutions and Food and Drug Administration guidelines, including patient consent where appropriate.

## Highlights

- Cardio-oncology patients account for an increasing proportion of echocardiography requests.
- Accurate assessment of LV systolic function is critical to decision making in this patient group.
- 2D LVEF, 3D LVEF, GLS, and RV assessment should be used in the echocardiographic assessment of these patients.
- The clinical implications of a significant decline in GLS with potentially cardiotoxic cancer therapy require further investigation.

Advances in cancer detection and treatment have resulted in a growing number of cancer survivors. Cardio-oncology is a relatively new subspecialty that aims to prevent, detect, monitor and treat the cardiac complications of cancer therapy (1). The goal of the cardio-oncologist is to provide optimal cardiovascular care for patients with cancer in a multi-disciplinary setting involving oncologists, cardiologists, surgeons, cardiac physiologists/scientists, specialist nurses, pharmacists, and allied health professionals (2). Cancer therapy-related cardiac dysfunction (CTRCD) is a frequently encountered clinical presentation, and transthoracic echocardiography is the cornerstone of its screening and detection.

The British Society of Echocardiography (BSE) has recently published an updated minimum dataset for a standard adult transthoracic echocardiogram (3). This cardio-oncology guideline is designed to be used in conjunction with the minimum dataset and provides guidance on transthoracic echocardiographic image acquisition and data interpretation in patients undergoing treatment with anthracyclines and/or trastuzumab.

This consensus guideline:

1. Defines the standard echocardiography protocol for the assessment of left ventricular (LV) function in those undergoing anthracyclines and/or human epidermal growth factor (EGF) receptor 2 (HER2)-targeted therapy.
2. Defines cardiotoxicity and specifically CTRCD with anthracyclines and/or HER2-targeted therapy.
3. Provides strategies to enable the acquisition of high-quality echocardiography for patients undergoing anthracyclines and/or HER2-targeted therapy.
4. Reviews the nonechocardiographic considerations for clinical decision making; reviews risk factors for cardiotoxicity; and provides guidance for referral to a cardio-oncology service.

## Background

Anthracyclines (e.g. doxorubicin, epirubicin, daunorubicin, and idarubicin) and the monoclonal antibody trastuzumab (Herceptin, Genentech, South San Francisco, California) are commonly implicated in the development of LV dysfunction (4). Although there are other cardiotoxic anticancer therapies, in our experience, patients receiving anthracyclines and/or trastuzumab account for the majority of cardio-oncology echocardiograms performed, hence are the focus of this guideline. Trastuzumab may also be prescribed in combination with pertuzumab, another HER2-positive-targeted monoclonal antibody, or with emtansine (Kadcyla/T-DM1, Genentech), which may be associated with additional cardiovascular concerns (5).

Many mechanisms are postulated to explain anthracycline-induced cardiotoxicity. Generation of excess reactive oxygen species and oxygen free radicals causing damage to DNA, RNA, proteins, and membrane lipids, and resultant cardiomyocyte death is one of the most commonly accepted cardiotoxicity mechanisms (6). The mechanisms responsible for trastuzumab-related cardiotoxicity are less clear but likely are related to inhibition of the neuregulin-1 (NRG-1)/ErbB signaling pathway (7). Commonly, but not in all cases, there is recovery of LV function with trastuzumab cardiotoxicity (8).

The addition of trastuzumab to anthracycline chemotherapy alone improves the overall survival of patients with HER2-positive tumors by approximately 33%, with a 50% reduction in disease recurrence (9, 10). For this reason, the management of cardiac dysfunction should first consider the initiation of cardioprotective therapies, rather than withholding prognostically important oncology treatment. Management decisions require close collaboration between oncology and cardiology specialists. In addition, the risk of cardiotoxicity is not just an issue during oncology treatment (chemotherapy and/or radiotherapy) but can remain a concern for many years thereafter (11, 12).

## The role of echocardiography and the recommended cardio-oncology protocol

All patients should undergo a comprehensive baseline echocardiogram to include the BSE minimum transthoracic dataset (3) with additional cardio-oncology measurements (Table 1). Best practice for the minimum dataset for a targeted cardio-oncology protocol includes 2D and 3D volumes, LV ejection fraction (LVEF), global longitudinal strain (GLS), right ventricular (RV) size and systolic function assessment, tricuspid regurgitant velocity (TRV), and blood pressure measurement (Table 2). Measurement techniques are described in Tables 1 and 2, and the overall clinical approach to echocardiographic monitoring is described in Figure 1.

## Baseline and serial echocardiographic assessment

The role of transthoracic echocardiography screening in the cardio-oncology setting is to assess cardiac function at baseline and to diagnose CTRCD at the earliest possible stage (Fig. 1). This enables informed decisions regarding timely commencement of cardioprotective medications and the safe continuation of cardiotoxic cancer therapy. It is crucial that accurate and reproducible parameters of LV systolic function are used so that a detected decline in LV systolic function truly reflects toxicity (13).

Baseline risk stratification of cardiotoxicity must take into consideration both the proposed cancer therapy and individual patient-related factors (Table 3). A more personalized tailored approach to surveillance is recommended in increased-risk patients compared with low-risk patients (Fig. 1). Recent Heart Failure Association–International Cardio-oncology Society expert position statements add to the published reports regarding the frequency of surveillance echocardiograms in patients stratified to low, medium, or high risk who then receive anthracyclines or trastuzumab (Table 4) (14, 15). In patients with normal LV systolic function at baseline, subsequent echocardiograms in asymptomatic patients should be targeted in the studies (Table 2). However, any patient with new cardiovascular symptoms while receiving cancer therapy should undergo a full echocardiogram (16).

The optimum frequency of echocardiograms during and after cancer therapy is unclear especially in the context of current pandemics (e.g. COVID-19) (17, 18). Recommendations for echocardiography during and after anthracycline-containing chemotherapy also differ, with the majority of guidelines not quantifying the frequency of monitoring (19, 20). There is a wide variation in the guideline recommendations (19) on the frequency of echocardiographic monitoring for patients receiving trastuzumab, ranging from every 3 months (21) to an undefined ‘periodically’ (10) (Table 4). Furthermore, there is no strong evidence to support a specific schedule of screening or any evidence that it improves outcomes for screened patients (22). However, screening every 3 months is still recommended by the US Food and Drug Administration, although the frequency is admittedly controversial, and the compliance is limited (23).

Historically, trastuzumab has been temporarily held or even discontinued in patients who develop LV systolic dysfunction. However, there are increasing data to suggest that patients with asymptomatic reductions in https://erp.bioscientifica.com
https://doi.org/10.1530/ERP-21-0001
© 2021 The authors
Published by Bioscientifica Ltd
This work is licensed under a Creative Commons Attribution-NonCommercial-NoDerivatives 4.0 International License.

**Table 1** Minimum requirements for baseline assessment for patients receiving anthracyclines/trastuzumab (in addition to the full BSE minimum dataset (3)).

| **View** (Modality) | **Measurement** | **Explanatory note** | **Image** |
|---|---|---|---|
| | Vital signs | Blood pressure, heart rate and rhythm | |
| Apical 3D | 3D volumes and LVEF | ECG signal with clear R-wave. Adjust scanner settings to ensure optimal resolution. Ensure ROI is within the 3D volume sector. Maximize the frame rate, adjusting number of subvolumes according to patient breath-holding capability as needed. Acquire images with the probe maintained in a steady position and at end-expiration. Before accepting acquisition, review volume and 9-slice view to ensure no stitch artifacts. |  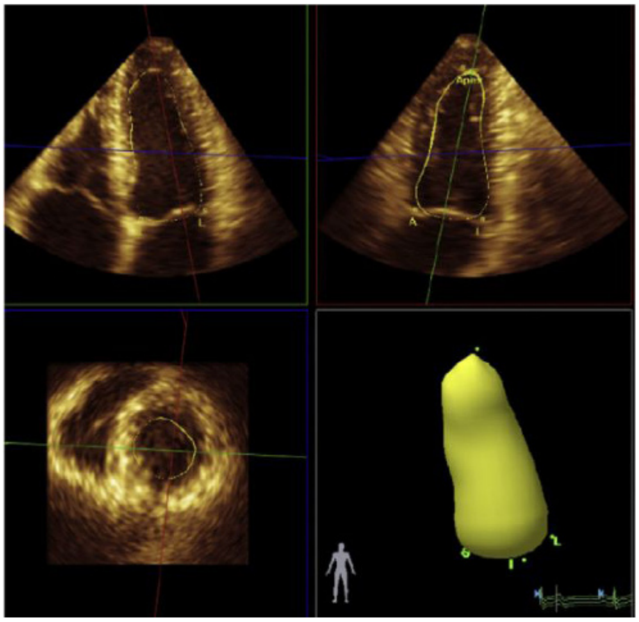 |
| A4C/A3C/A2C GLS | GLS measurement | Optimal ECG signal with minimal heart rate variability should be present across the three cardiac cycles. Heart rate variability will limit the calculation of GLS values, which can be problematic in patients with atrial fibrillation. High-quality image acquisition, maintaining a frame rate of 40 to 90 frames/s at a normal heart rate is key. |  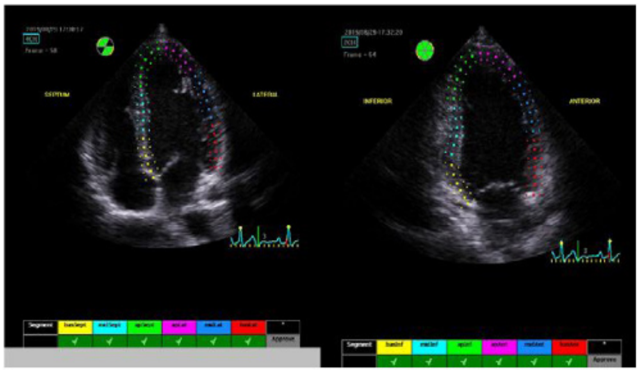 |
| | | Clear endocardial and epicardial definition is required to ensure adequate segmental tracking throughout the cardiac cycle. Markers are placed in each of the respective basal and apical regions, using automated tracking where possible to maintain reproducible results. Automated tracking should also be combined with a visual assessment of tracking in each view across the whole ROI, including the endocardial and epicardial border. If more than two segments in any one view are not adequately tracked, the calculation of GLS should be avoided. |  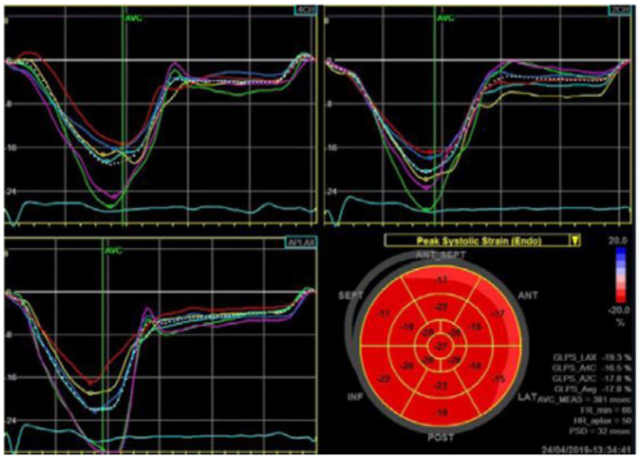 |

3D, 3-dimensional; A2C, apical 2 chamber; A3C, apical 3 chamber; A4C, apical 4 chamber; BSE, British Society of Echocardiography; GLS, global longitudinal strain; LVEF, left ventricular ejection fraction; ROI, region of interest.

**Table 2** Cardio-oncology targeted echocardiogram reporting protocol.

| **View** (modality) | **Measurement** | **Explanatory note** | **Image** |
|---|---|---|---|
| | Vital signs | Blood pressure, heart rate and rhythm | |
| A4C and A2C 2D | Simpson's biplane volumes and LVEF | Trace the endocardial border. Depending on the vendor, the MV level contour is made by a straight line at the beginning or end of tracing. LV length is defined as the distance between the mid-point of the MV-level line and the most distal point of the LV apex. Take care to ensure the LV is not foreshortened. Papillary muscles and trabeculations are included in the volumes and considered part of the chamber. Measure at end-diastole and end-systole. Volumes indexed to BSA. |   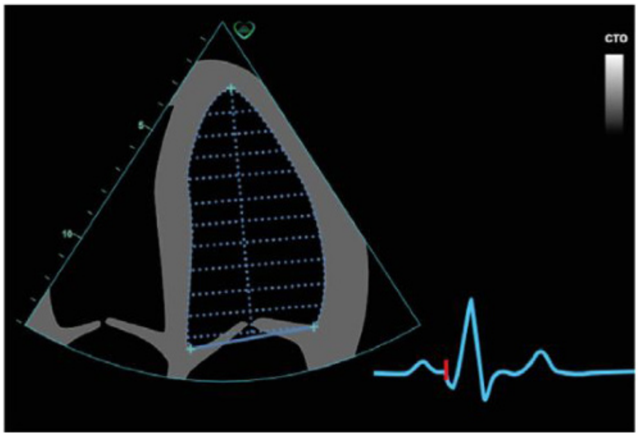 |
| Apical 3D | 3D volumes and LVEF | See Table 1 | |
| A4C/A3C/A2C | GLS | See Table 1 | |
| A4C LV TDI | S′ | Place sample volume (5 to 10 mm) at or within 1 cm of the insertion of the MV leaflets. Angle of interrogation should be as parallel to Doppler beam as possible. Measure at end-expiration. Optimize scale and sweep speed (100 mm/s). Average both septum and lateral wall measurement. S′: peak systolic velocity. |   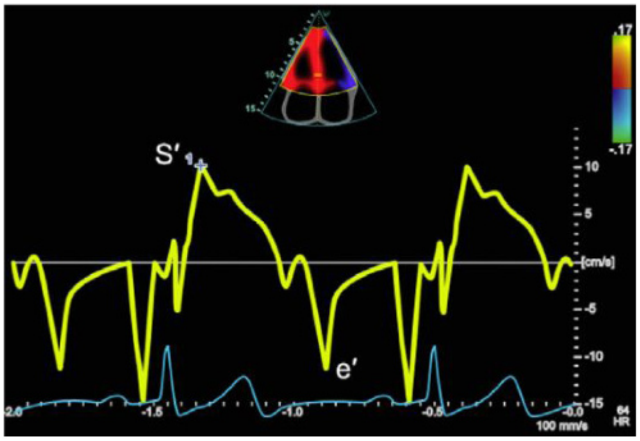 |
| Modified A4C RV (2D) | RVD1 (±RVD2/RVD3) | RVD1: basal RV diameter. Measured at the maximal transverse diameter in the basal one-third of the RV. RVD2: mid-RV diameter measured at the level of the LV papillary muscles. RVD3: RV length, from the plane of the tricuspid annulus to the RV apex. |   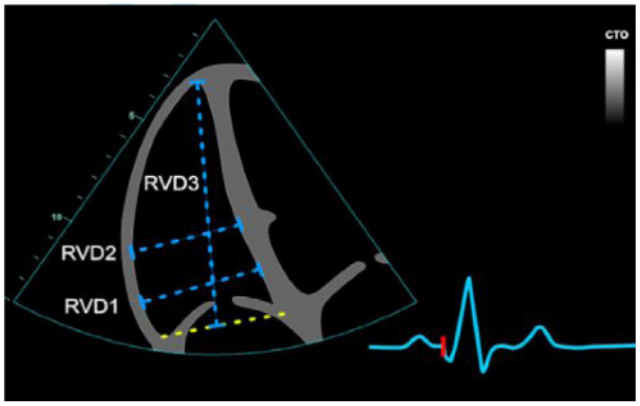 |

*(Continued)*

© 2021 The authors
Published by Bioscientifica Ltd
This work is licensed under a Creative Commons Attribution-NonCommercial-NoDerivatives 4.0 International License.

**Table 2** Continued.

| **View** (modality) | **Measurement** | **Explanatory note** | **Image** |
|---|---|---|---|
| CWD TV | TR peak velocity ($TRV_{max}$) | Peak TR velocity is measured by CWD across the tricuspid valve. Ensure the CWD to flow angle is correctly aligned. Eccentric jets can lead to incomplete Doppler envelopes and underestimation of TR velocity. A high sweep speed (100 mm/s) can help to differentiate between true velocities and artifact. Measure from a complete TR envelope. Choose the highest velocity. Accuracy is greatest when ultrasound and blood flow are parallel. |   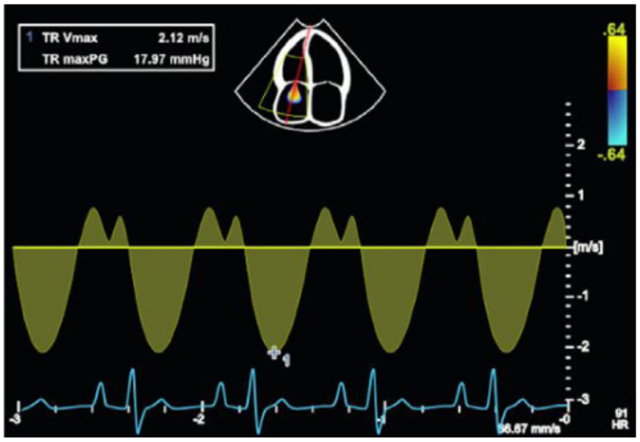 |
| A4C RV (TDI) | RV S′ | PW tissue Doppler S′ wave measurement taken at the lateral tricuspid annulus in systole. It is important to ensure the basal RV free wall segment and the lateral tricuspid annulus are aligned with the Doppler cursor to avoid velocity underestimation. A disadvantage of this measure is that it assumes that the function of a single segment represents the entire function of the entire ventricle, which is not likely in conditions that include regionality such as RV infarction. Normal value ≥ 9 cm/s (27). |   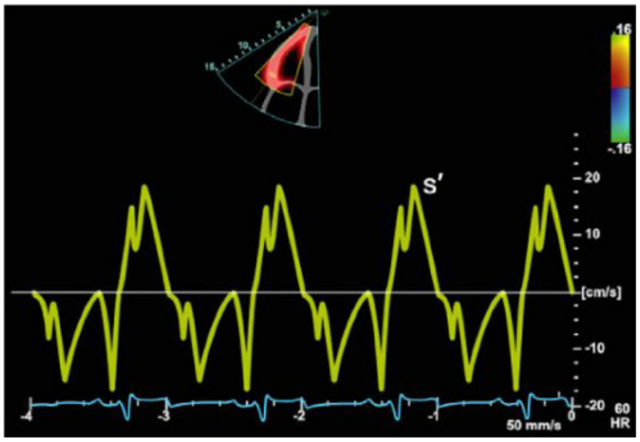 |
| A4C lateral TV annulus (MM) | TAPSE | This is an angle-dependent measurement, and therefore, it is important to align the M-Mode cursor along the direction of the lateral tricuspid or mitral annulus. Select a fast sweep speed. Measure total excursion of the tricuspid annulus. Normal value ≥ 17 mm (60). |   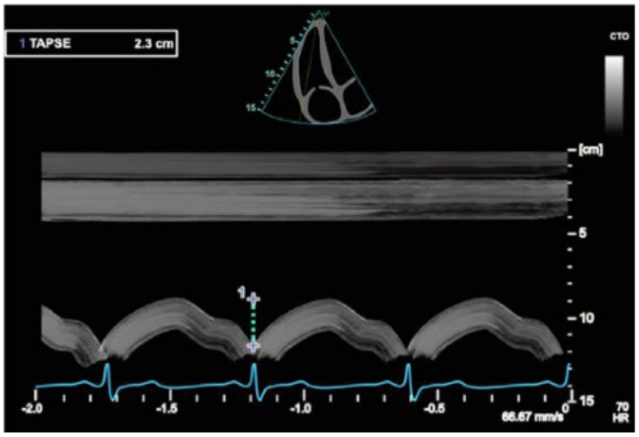 |

BSA, body surface area; CWD, continuous-wave Doppler; LA, left atrium; LV, left ventricle; MM, M-mode; MV, mitral valve; PW, pulsed wave; RV, right ventricle; RVD, right ventricular diameter; TAPSE, tricuspid annular plane systolic excursion; TDI, tissue Doppler imaging; TR, tricuspid regurgitation; TV, tricuspid valve; other abbreviations as in Table 1.

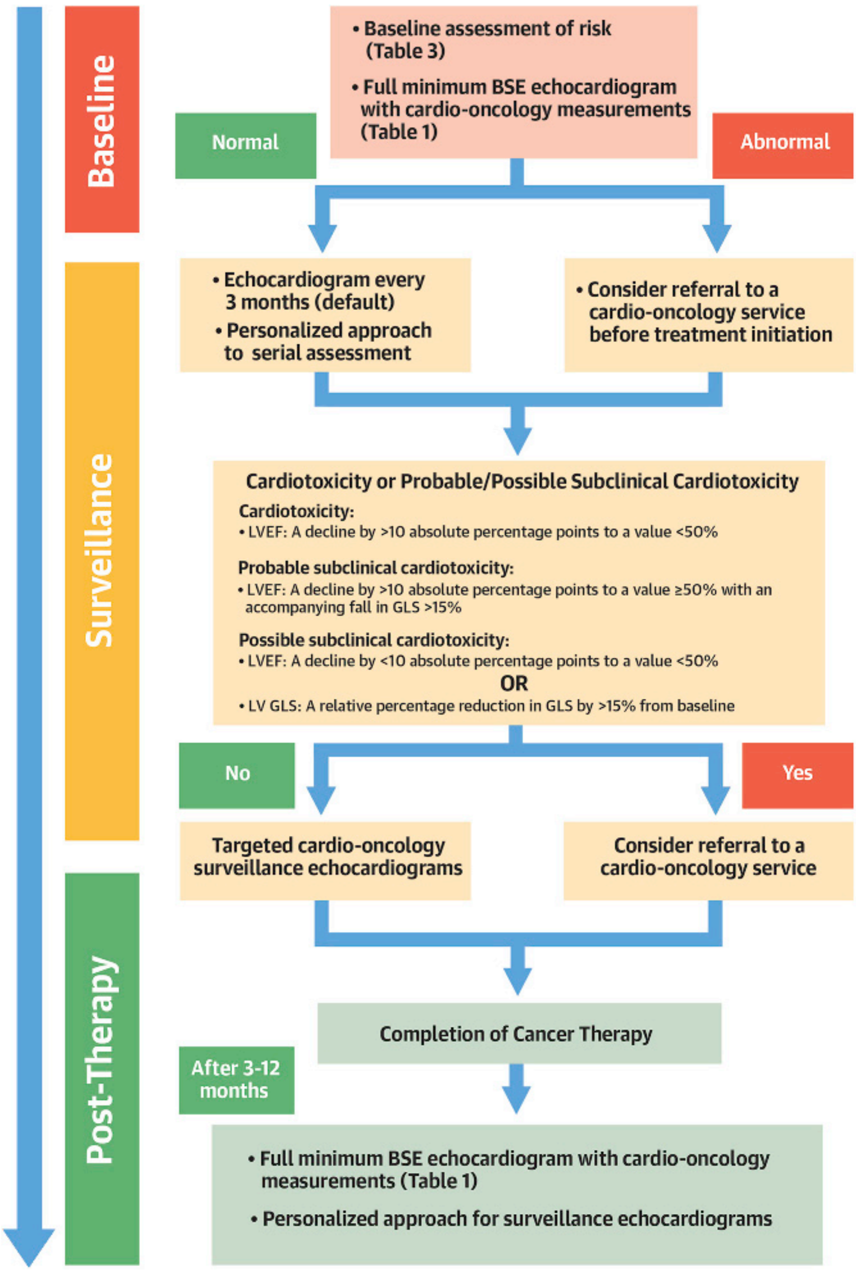

**Figure 1**
Echocardiography protocol in patients undergoing treatment with anthracyclines/HER2-positive-targeted therapy. Assessment at baseline, during therapy (including patients on indefinite HER2-positive-targeted therapy in case of metastatic disease) and long-term follow-up after the completion of cancer therapy. BSE, British Society of Echocardiography; GLS, global longitudinal strain; LV, left ventricular; LVEF, left ventricular ejection fraction.

LVEF to 40 to 49%, with guidance from a cardio-oncology team and personalized monitoring and treatment, can safely complete their cancer treatment without a significant increase in cardiac events (24, 25). We, therefore, recommend a personalized approach to patient surveillance, as emphasized in a position statement from the Heart Failure Association–European Association of Cardiovascular Imaging (15).

Echocardiography 3 to 12 months post-cardiotoxic treatment is recommended in all the patients, with the optimum timing dependent upon the individual patient's risk (16). Appropriate frequency of repeat echocardiography thereafter remains to be fully defined and depends upon whether any cardiotoxicity occurred during the treatment phase (15), with international recommendations varying from 1- to 5-year intervals (16, 26). Decisions regarding long-term surveillance should take into consideration a patient's total anthracycline dose, exposure to other potentially cardiotoxic treatments (including radiotherapy), cardiovascular comorbidities, cardiotoxicity during treatment, and LV systolic function during and at the end of treatment.

**Table 3** Identification of the patient at increased risk of cardiotoxicity.

| Lower risk | Increased risk |
|---|---|
| **Therapy-related risk factors** | |
| Lower lifetime dose of anthracycline | Increased lifetime dose of anthracycline |
| < Doxorubicin 250 mg/m$^2$ or equivalent | > Doxorubicin 250 mg/m$^2$ or equivalent – high risk |
| No previous anthracycline/trastuzumab-related cardiotoxicity | > 400 mg/m$^2$ or equivalent – very high risk |
| Absence of sequential anthracycline and trastuzumab therapy | Prior anthracycline/trastuzumab-related cardiotoxicity |
| Low-dose radiation therapy to central chest including heart in radiation field < 30 Gy | Sequential anthracycline and trastuzumab therapy |
| | High-dose radiation therapy to central chest including heart in radiation field ≥ 30 Gy |
| **Patient-related risk factors** | |
| Male | Female |
| Age < 50 years | Age 50 to 64 years – high risk and ≥ 65 years – highest risk |
| Absence of traditional cardiovascular risk factors: hypertension, smoking, obesity, dyslipidemia, insulin resistance | Presence of traditional cardiovascular risk factors: hypertension, smoking, obesity, dyslipidemia, insulin resistance |
| Past medical history: | Past medical history: |
| Normal baseline LVEF | Reduced or low-normal LVEF (50 to 54%) pre-treatment |
| Absence of pre-existing cardiovascular disease (e.g. CAD, PAD, cardiomyopathy, severe valvular heart disease, heart failure, or diabetes) | Presence of pre-existing cardiovascular disease (e.g. CAD, PAD, cardiomyopathy, severe valvular heart disease, heart failure, or diabetes) |
| Normal kidney function or chronic kidney disease stage 1 | Chronic kidney disease stage 2 + (eGFR < 78 mL/min/1.73 m$^2$) (84) |
| Biomarkers: | Biomarkers: |
| Normal baseline troponin and/or NT-proBNP | Elevated* baseline troponin and/or NT-proBNP |
| Normal cardiac troponin or NT-proBNP during cancer therapy | Elevated* cardiac troponin or NT-proBNP during cancer therapy |

*Elevated above the upper limit of normal for local laboratory reference range.
CAD, coronary artery disease; eGFR, estimated glomerular filtration rate; LVEF, left ventricular ejection fraction; NT-proBNP, N-terminal pro-B-type natriuretic peptide; PAD, peripheral arterial disease.

## Echocardiography-based definitions of cardiotoxicity

The definition of cardiotoxicity is varied and not limited to LV systolic dysfunction and CTRCD (19). The definition of cardiotoxicity based solely on LVEF also varies significantly (10, 16, 21). We define CTRCD as a decrease in LVEF by > 10% (10 absolute percentage points) to a value < 50%. This is in keeping with BSE-published normal/borderline normal ranges (27) and European Society for Medical Oncology consensus recommendations (26). A LVEF of 50 to 54% is considered to be borderline low and will require more information before labeling the patient as having normal or abnormal LV systolic function.

We recommend that, if possible, 3D LVEF is measured due to both its reported superior reproducibility compared with 2D LVEF in patients undergoing anticancer therapy (28) and suggestions that 3D LVEF changes are more pronounced than and precede 2D LVEF changes in such patients (29). 3D LVEF has been shown to allow accurate serial quantification of LV systolic function and identification of changes in oncology patients (13). Decline in LVEF are usually accompanied by a significant change in GLS. Therefore, if GLS is normal in the presence of a reduced LVEF, a review of the echocardiograms to reassess the accuracy of all measurements is recommended. If there is still a significant and unexplained discrepancy between change in LVEF and GLS, adjudication with cardiac magnetic resonance (CMR) imaging should be considered.

Normal GLS values vary with age, sex, loading conditions, and different vendors; therefore, definition of abnormal GLS is not straightforward. It is important that heart rate and blood pressure are recorded because variation will need to be considered if there are temporal changes in GLS measurements. Much of the existing published reports on GLS values are based on General Electric vendor-specific data, and for the purpose of this guideline, we define a normal GLS value as being −17% or more negative for males and −18% or more negative for females (30, 31, 32). A relative change in sequential GLS > 15% (e.g. −22% to −18%) is considered to be significant (33). A worsening in GLS is known to predict a subsequent decline in LVEF, with GLS-guided cardioprotective therapy potentially reducing a decline in LVEF (34). The change in GLS is essential in recognition of cardiotoxicity, such that each patient acts as their own control. For this reason, comprehensive baseline echocardiography before cancer therapy is critical.

Reduction in GLS into the abnormal range as described or borderline values and decline in LVEF within the normal range should not be taken in isolation, especially in asymptomatic patients, but be interpreted in the overall clinical context. A decline in LVEF by > 10% points to an absolute value > 50% with a lower limit of

© 2021 The authors
Published by Bioscientifica Ltd
This work is licensed under a Creative Commons Attribution-NonCommercial-NoDerivatives 4.0 International License.

**Table 4** Frequency of echocardiographic monitoring during anthracycline or trastuzumab (Anti-HER2) therapy according to the published guidelines.

| **Guideline, year** (Ref. #) | **Recommendation for frequency of echocardiography during therapy** |
|---|---|
| HFA-EACVI, 2020 (15) | |
| Anthracyclines | Low risk*: after cycle of cumulative dose 240 mg/m² doxorubicin or equivalent, then every additional 100 mg/m² or every two cycles<br>Medium risk*: following 50% of planned total treatment and after cycle of cumulative dose 240 mg/m² doxorubicin or equivalent<br>High risk*: every two cycles, consider after every cycle above 240 mg/m² doxorubicin or equivalent |
| Anti-HER2 (neoadjuvant and adjuvant) | Low risk*: every four cycles (12 weeks)<br>Medium risk*: every three cycles (9 weeks), then reduce to every four cycles if stable at 4 months<br>High risk*: every two cycles (6 weeks), then reduce to every three cycles if stable at 4 months |
| Anti-HER2 (long term) | Low risk*: every four cycles in year 1, every six cycles in year 2, then reduce to every 6 months<br>Medium risk*: every three cycles, then if stable reduce to every 6 months<br>High risk*: every two or three cycles for 3 months, then reduce to every four cycles in year 1, then reduce frequency |
| ESMO, 2020 (26) | |
| Anthracyclines | After a cumulative dose of 250 mg/m² doxorubicin or equivalent, then after each additional 100 mg/m² |
| Anti-HER2 | Every 3 months (higher-risk patients may require more frequent monitoring) |
| Anti-HER2 (long term) | General surveillance, which may include cardiac imaging |
| ASCO, 2017 (16) | |
| Anthracyclines | Frequency of surveillance should be determined by health care providers; routine surveillance imaging may be offered in patients considered to be at increased risk of cardiac dysfunction |
| Anti-HER2 | Frequency of surveillance should be determined by health care providers; routine surveillance imaging may be offered in patients considered to be at increased risk of cardiac dysfunction |
| CCS, 2016 (85) | |
| Anthracyclines | No recommendation made |
| Anti-HER2 | Every 3 months |
| ESC, 2016 (10) | |
| Anthracyclines | After 200 mg/m² of doxorubicin or equivalent |
| Anti-HER2 | Every four cycles |
| ASE, 2014 (33) | |
| Anthracyclines | After 240 mg/m² of doxorubicin or equivalent, then after each additional 50 mg/m² |
| Anti-HER2 | Every 3 months |

*Risk is calculated according to therapy and patient-related factors, including age, and cardiovascular risk factors. For more details, the reader is directed to the original guideline (15).

ASCO, American Society of Clinical Oncology; ASE, American Society of Echocardiography; CCS, Canadian Cardiovascular Society; EACVI, European Association of Cardiovascular Imaging; ESC, European Society of Cardiology; ESMO, European Society of Medical Oncology; HER, human epidermal growth factor; HFA, Heart Failure Association.

normal of GLS is a gray area that might suggest potential subclinical cardiotoxicity, although in some cases, it may reflect normal, physiological variability (35). In these circumstances, it is important to take other factors (such as symptoms, other echocardiographic parameters of LV systolic function, and biomarkers) into consideration. GLS may have added value, particularly in cases of borderline LVEF, with a normal strain measurement providing some reassurance (36, 37). Patients with normal LVEF, but abnormally low strain, also require further investigation to rule out potential causes such as cardiac amyloidosis, infiltration or hypertensive cardiomyopathy (38). These echocardiograms should be reviewed (to also ensure adequate technical quality) in conjunction with a clinical assessment and biomarker measurement. Certainly, there is a need for longer-term data to define the natural history of LVEF and GLS changes as well as the prognostic implications of potential subclinical cardiotoxicity.

## Recommendations

Definition of cardiotoxicity by echocardiography:

- LVEF: a decline in LVEF by > 10% (absolute percentage points) to a value < 50%,

Definition of probable subclinical cardiotoxicity by echocardiography:

- LVEF: a decline in LVEF by > 10% (absolute percentage points) to a value ≥ 50% with an accompanying fall in GLS > 15% (where GLS measurement is available).

Definition of possible subclinical cardiotoxicity by echocardiography:

- LVEF: a decline in LVEF by < 10% (absolute percentage points) to a value < 50%.

or

- LV GLS: a relative percentage reduction in GLS by > 15% from the baseline value.

The detection of cardiotoxicity or probable/possible subclinical cardiotoxicity is achieved via advanced echocardiographic measures (2D/3D LVEF and GLS). For best practice, centers undertaking surveillance of these patients should have the ability to perform 2D/3D LVEF and GLS assessment.

## LV function assessment by echocardiography

### Cardiac rhythm and rate

Sequential assessments for those not in sinus rhythm may be problematic. In atrial fibrillation due to the persistent variation in cardiac cycle length, measures of ventricular systolic and diastolic function may have limited reliability. When preceding and pre-preceding RR intervals are within 60 ms of each other and both exceed 500 ms, measures of systolic function on a single beat are similar to those averaged over 15 cycles of varying durations (39). These findings suggest that selection of beats with similar RR intervals is more important for reproducibility than the total number of measurements made.

### Blood pressure

Recording of blood pressure is essential because parameters such as LVEF, tissue Doppler indices, and GLS are load dependent. A substantial increment of blood pressure may be responsible for an apparent change in function, without necessarily indicating myocardial disease, because poorly controlled hypertension is associated with abnormal strain (40).

### Accurate assessment of 2D LVEF

LVEF is one of the most commonly used echocardiographic methods to assess LV systolic function. This represents the fraction of blood within the LV that is ejected in one cardiac cycle. Because it is difficult to quantify a 3D structure using 2D imaging, the techniques developed with 2D echocardiography rely on measuring the ventricle in standard planes.

We do not support the use of qualitative ‘eyeball’ assessments to determine LVEF values or ranges. The 2D volumetric Simpson’s method for the assessment of LVEF is based on the principle of slicing the LV from the apex down to the mitral valve (MV) into a series of discs. The volume of each disc is then calculated using the diameter and thickness of each slice. It is assumed that the LV is circular at each level. Accuracy is improved by using diameters in two planes, separated by 60° of rotation (apical 4 and 2 chamber) so that the disc surface area is more precisely defined. Acquisitions should be made at end-respiration, and the image should be adjusted using gain, compress, and dynamic range to ensure optimal endocardial delineation and elongation of the entire LV length, without foreshortening. High-quality images are critical for accurate quantification. For more detailed guidance, the reader is directed to the BSE minimum dataset (3). The endocardial surface should be traced at end-diastole and end-systole to encompass the whole of the LV. Papillary muscles and trabeculations are excluded from the endocardial tracing and are considered part of the chamber. This can be achieved either on the machine or by using an off-line analysis software package. LVEF is calculated as the difference between end-diastolic volume and end-systolic volume (stroke volume) as a percentage of the end-diastolic volume. Intraobserver and interobserver variability has been reported as 3.3 and 4%, respectively with a minimum detectable difference of 9 to 11% (13, 41). When describing 2D LVEF, the report conclusion should always note the most recent estimate of LVEF and include a comparison with the baseline value.

### 3D LVEF

The 2D echocardiographic assessment of LVEF has inherent limitations because it makes geometric assumptions of the LV. This consideration is especially important for serial echocardiograms because exact plane duplication is almost impossible. 3D echocardiography, although still requiring high-quality, reproducible images, is an advance on the Simpson’s method because it allows contouring of the cavity within the 3D space of the echocardiographic volume acquisition. Therefore, there is no assumption that the short-axis view of the ventricle is circular. Instead, one can contour the actual shape of the ventricle in all dimensions. Consequently, 3D LVEF calculation has a smaller detected percent change of approximately 5 to 8% and, because it is semiautomated, has better intra- and interobserver variability (13). There is superior temporal variation of 3D LVEF compared with 2D LVEF over a 12-month period in stable patients receiving chemotherapy (3% vs 5%) (13). Commercially available scanners contain software tools that allow the 3D assessment of LV volumes and LVEF. It is to be remembered, however, that 3D imaging remains sensitive to image quality, and it is good practice to record and analyze both 2D and 3D images, because deterioration of image quality during follow-up is most likely to affect 3D images. There remains some variation in 3D LVEF calculation between specific vendors, and we recommend that the same machine and analysis software are used for serial echocardiograms (42). When reporting 3D LVEF findings, the conclusion should always note the most recent estimate of LVEF and should include a comparison to the baseline value.

Full-volume datasets allow the generation of a 3D dataset with the final image of the heart created by acquiring several single or several subvolumes over the corresponding number of sequential cardiac cycles. Attention to breath-hold is essential to avoid stitching artifact. The greater the number of subvolumes used, the higher the frame rate and temporal resolution, albeit at greater risk of stitching artifact. Newer technology allows increased volume rates and reduced time for complete cardiac volume acquisition in the estimation of 3D LVEF, making it comparable to CMR imaging (43). It should be recognized that there is a trade-off between temporal and spatial resolution. Temporal resolution (i.e. volume or frame rate) allows localization of an anatomic structure in a point in time. This can be improved by reducing the sector size (width and depth). Spatial resolution is the ability to differentiate two points in space and is dependent on the number of scan lines per volume (scan line density), However, increasing scan lines lengthens the acquisition time and lowers the volume rate (44).

Images should be obtained during shallow breathing, preferably in end-expiration. If deep inspiration is needed to obtain optimal image quality, this should be documented in the echocardiogram report so that it can be reproduced at the next visit. Finally, it is good practice for the sonographer to quantify LVEF on 2D and 3D images while the patient is still present. Performing the analysis at the same time also allows repeat image acquisition in case of contouring difficulty.

How to acquire 3D echo volumes for LVEF assessment:

- Ensure high-quality ECG trace with a clear R-wave. This enables appropriate 3D full-volume triggering.
- Ensure the region of interest (ROI) is within the 3D volume sector. Reduce the sector as needed to focus on the ROI.
- As for 2D imaging, adjust scanner settings so that the best 3D resolution is available.
- Adjust gain appropriately. Low gain settings result in echo ‘dropout’, and excess gain reduces resolution and causes a loss of the 3D perspective or depth in the dataset.
- Optimize frame rate and adjust number of subvolumes according to the patient’s breath-holding capacity as needed.
- Acquire images with the probe maintained in a steady position and at end-expiration.
- Following acquisition, review the image to look for any stitch artifact.

## Contrast echocardiography/ultrasound enhancing agents

Inadequate LV endocardial border definition can lead to errors in LV volume and LVEF estimation. Accurate LVEF assessment is particularly important where values obtained fall on boundaries that influence treatment decisions. Poor endocardial definition can occur in patients undergoing cancer treatment (e.g. following mastectomy, chest irradiation, or breast reconstruction surgery) (33) or secondary to body habitus. The use of echocardiographic contrast for LV chamber opacification is now widely accepted when two contiguous LV segments from any apical view are not adequately visualized on noncontrast images (45). Tracing LV borders more reliably leads to inclusion of LV trabeculation within the LV cavity after contrast. As a result, LV volumes (both in systole and diastole) are commonly greater than those recorded with noncontrast imaging, although LVEF is usually analogous. The minimum detectable difference for 2D contrast LVEF has been noted to be in the order of 4% (46), which is significantly better than the 9 to 11% for noncontrast 2D LVEF. However, the superior performance of LV contrast has not been consistently proven. One study demonstrated inferior reproducibility with contrast echocardiograms compared with noncontrast 2D and 3D echocardiograms (13). Use of the same methodology in sequential testing is thus recommended. It is to be noted that the use of contrast has unpredictable effects on 2D speckle tracking and is best done after strain acquisition.

## Tissue Doppler assessment of systolic and diastolic function

Tissue Doppler echocardiography has become an established component of the diagnostic ultrasound examination, enhancing interrogation of myocardial motion. Although LVEF reflects the sum contribution of several regions, it does not provide information on regional function or on the underlying myocardial mechanical activity. Conventional Doppler techniques assess velocity of blood flow by measuring high-frequency, low-amplitude signals from small, fast-moving blood cells. Tissue Doppler imaging (TDI) uses the same Doppler principles to quantify the higher-amplitude, lower-velocity signals of myocardial tissue motion. TDI depicts myocardial motion at a specific location in the heart. High-velocity signals from the blood are filtered out and amplification scales suitably adjusted so that Doppler signals from tissue motion can be recorded. Tissue velocity indicates the rate at which a particular point in the myocardium moves toward or away from the transducer. The accuracy of TDI is angle dependent and only measures the vector of motion that is parallel to the direction of the ultrasound beam. Mean mitral annular S′ should be acquired at end expiration. For more detailed guidance, the reader is directed to the BSE minimum dataset (3). Normal age-related values for mean mitral annular S′ are described in recent guidance (27). Both tissue Doppler and gray scale imaging have been used to calculate mitral annular plane displacement, which is a longitudinal function parameter analogous to strain.

In breast cancer patients receiving anthracyclines with or without trastuzumab, diastolic dysfunction has been reported to precede systolic dysfunction and CTRCD (47). We recommend that diastolic assessment should be undertaken in all baseline echocardiograms. Along with mitral E and A maximum velocity (Vmax), E/A ratio, left atrial volumes, and TRV, TDI is a key part of the assessment of diastolic function (48). For more details on the grading of diastolic function, the reader is directed to the current American Society of Echocardiography/European Association of Cardiovascular Imaging guidelines on the assessment of diastolic dysfunction (49).

## Speckle tracking echocardiography GLS

The term *strain* refers to an object's fractional or percentage change from its original, unstressed dimension and reflects the deformation of a structure. When applied to myocardium, this deformation or strain directly describes the contraction/relaxation pattern. At rest, an object that has an initial length ($L_0$) can be stretched or compressed to a new length (L). This change in length is usually represented as a percentage, with a negative score indicating a shortening in length. If L equals $L_0$, then strain remains zero.

Although LVEF is simple and intuitive, and supported by prognostic information, it has important limitations including image quality dependence, geometric assumptions, and insensitivity to early disease (which is characterized by disturbances of longitudinal function). Strain measurements, like LVEF measurements, are dependent on endocardial border tracing and therefore also rely on image quality.

The myocardial fiber orientation of the LV is complex. The major limitation of the Doppler-based approach is the angle dependency required during image acquisition (50, 51). This has been overcome by speckle tracking echocardiography, which is based on tracking the pattern of speckles generated by reflected ultrasound signal. Different regions of myocardium have a unique speckle pattern that moves from one frame to the next. Dedicated speckle tracking software enables this movement to be quantified via several parameters (such as longitudinal strain).

GLS is measured using a combination of the apical 2-chamber (A2C), 3-chamber (A3C), and 4-chamber (A4C) views (52). Longitudinal strain is the degree of deformation from base to apex. During systole, contraction in this plane leads to fiber shortening, represented as a negative percentage value (i.e. the more negative the value, the greater the deformation). Although global circumferential and radial strain may also indicate cardiotoxicity, there are less data to support their clinical use (35); hence, the focus on GLS. GLS has been shown to be superior to 2D LVEF with regard to reproducibility in patients receiving trastuzumab (53) and has been suggested that it is more reproducible with appropriate echocardiographic training (54). It is therefore best practice that cardio-oncology echocardiograms are performed on machines that are able to calculate GLS and which have 3D capabilities. Small, but statistically significant, differences between vendors exist; therefore, the same acquisition platform and analysis software should be used for serial echocardiograms (55, 56, 57). The ability to perform GLS and 3D measurements is beyond the current BSE personal accreditation standards. It is, therefore, important that individuals undertaking cardio-oncology echocardiograms are suitably trained in acquisition and analysis of these advanced echocardiography measures in a reproducible and consistent manner.

In addition, echo departments should establish and reassess their intra- and interobserver variability of 2D LVEF, 3D LVEF, and GLS.

How to perform GLS:

- GLS is calculated using the standard A3C, A4C, and A2C views.
- Ensure that an optimal ECG signal with minimal heart rate variability is present across the three cardiac cycles.
- Maintain a frame rate of 40 to 90 frames/s (33) at a normal heart rate.
- Focus on the LV with appropriate adjustment of width and depth.
- The technique used to select the appropriate ROI is vendor specific, and the reader is advised to consult individual machine/software technical guidelines for further guidance. For General Electric machines/software, adjust the overlay for the ROI. In the A4C view, the ROI begins at the septal MV annulus, progresses to the apex, and ends at the lateral MV annulus. In the A2C view, the ROI starts at the inferior MV annulus and extends through to the apex and then to the anterior wall MV annulus. In the A3C view, the ROI starts at the posterior wall MV annulus, extends to the apex and finally to the base of the septal wall, taking care not to extend into the LV outflow tract.
- Two contours for speckle tracking are visible and should be aligned with the relevant area of interest:
  - The endocardial border – the inner contour of the myocardium.
  - The epicardial border – the outer border of the myocardium (be careful to exclude the pericardium, especially if automated analysis software is used. Inclusion of pericardium will lead to an underestimation of strain).
- Use optimal gain settings and breath-holding techniques to clearly delineate the endocardial and epicardial borders.
- During post-processing, the ROI should be aligned as accurately as possible to reflect the 17-segment LV model.

### Right heart assessment

Right heart (RH) structure and function have not traditionally been incorporated into the definition of CTRCD. However, there is increasing evidence that RH abnormalities may be prognostically significant (58, 59), and, therefore, assessment of the RH should be obtained to include RV dimensions, RV S′, tricuspid annular plane systolic excursion, and TRV. Abnormalities in RH structure and function on serial echocardiography should be discussed with a cardio-oncologist and may require full assessment of the RH as per the BSE practical guideline for RH assessment and the BSE guideline on the assessment of pulmonary hypertension (60, 61).

## Alternative imaging modalities

Although echocardiography remains the first-line investigation for the detection of CTRCD, there is a complementary role for other imaging modalities, particularly in patients with inadequate echocardiographic windows. Traditionally, multigated acquisition scans have been used in the assessment of LV systolic function, however, the associated radiation exposure (a particular issue with the inevitable serial scans) and limited structural information available makes this an inferior investigation (62). CMR imaging may be required in patients with poor echocardiographic windows or for tissue characterization (e.g. cardiac masses and cancer treatment-related myocarditis). Low inter- and intrareader variability make it the optimal technique for detecting small changes in LVEF in serial scans; however, its use is still limited by its availability and cost (63, 64). The role of cardiac CT is mainly in the noninvasive evaluation of coronary artery disease, but it can also be used to assess pericardial disease and valvular heart disease and in the imaging of cardiac tumors (65, 66).

### Recommendation

- Contrast 2D echocardiography should be considered when subendocardial definition precludes the accurate assessment of LVEF, that is, when a minimum of two contiguous LV segments from any apical view are not seen on noncontrast images (45).
- Depending on local expertise and availability, CMR imaging is an alternative modality, particularly for patients with poor echocardiographic windows.
- The same imaging modality should be used for sequential scans.

## Clinical risk stratification for cardiotoxicity

Risk stratification for cardiac dysfunction is recommended before the commencement of potentially cardiotoxic cancer treatment in all patients (14, 16). A clinical history

**Table 5** Key echocardiographic recommendations for best practice.

| | |
|---|---|
| Baseline assessment | Full BSE minimum dataset echocardiogram, vital signs, and GLS/3D volumes (see Table 1) |
| Follow-up assessment | Targeted echocardiogram (see Table 2).<br>If new symptoms, then full echocardiogram as per baseline assessment |
| Definition of cardiotoxicity | LVEF: a decrease in LVEF by >10% (absolute percentage points) to a value < 50% |
| Definition of probable cardiotoxicity by echocardiography | LVEF: decrease in LVEF by >10% (absolute percentage points) to a value ≥ 50% with an accompanying fall in GLS > 15% (where GLS measurement available) |
| Definition of possible cardiotoxicity by echocardiography | LVEF: a decrease in LVEF by <10% (absolute percentage points) to a value < 50% or LV GLS: when LVEF ≥ 50%, a relative percentage reduction in GLS by > 15% |
| Poor endocardial definition | Consider contrast echocardiography when endocardial definition precludes the accurate assessment of LVEF (e.g. when a minimum of two contiguous LV segments from any apical view are not seen on noncontrast images)<br>Depending on local expertise and availability, CMR imaging is an alternative modality in this context |

CMR, cardiac magnetic resonance; other abbreviations as in Tables 1, 2 and 3.

and cardiovascular examination, including blood pressure measurement, should be performed in order to aid assessment of risk (Table 3). All patients should also have a baseline 12-lead ECG.

Although it is outside the scope of this document, biomarkers (troponin and N-terminal pro-B-type natriuretic peptide (NT-proBNP)) may be considered in high-risk patients to assist further risk stratification (Table 3). Biomarkers may be able to detect subclinical signs of LV systolic and diastolic dysfunction before a decline in LVEF (67). Elevated baseline levels should prompt more frequent monitoring because there is some evidence that abnormal baseline high-sensitivity troponin is associated with an increased risk of developing complications with chemotherapy. There is also an association between troponin release after high-dose chemotherapy and subsequent cardiac events (68, 69, 70). Persistent serial elevation of NT-proBNP is associated with an increased risk of developing overt heart failure, whereas a transient rise is not (71). However, discussion of the utility and prognostic value of biomarkers is beyond the scope of this guideline, and the reader is directed to additional references (27, 72).

Defining the high-risk patient is challenging. Although there is no validated unifying risk calculator that is applicable to all cancer types and therapies (73), recently published guidelines can help clinicians with risk stratification (14). This highlights the need for a tailored and individualized approach to the assessment, treatment, and monitoring of cardio-oncology patients.

## Referral to a cardio-oncology service

The cardio-oncology team is composed of specialized health care professionals who work together to provide 'consistent, continuous, coordinated and cost-effective care during the cancer process' (74). This highly specialized service is involved in patient care, cardio-oncology research, and regional co-ordination of services (75).

Echocardiography is pivotal to decision making in cardio-oncology, for example, when to consider referral to a cardio-oncologist, when to initiate cardioprotective medications or heart failure therapy, and when to hold or discontinue cardiotoxic cancer therapy. Early detection of cardiac dysfunction, with prompt initiation of cardioprotective medications, increases the likelihood of LVEF recovery and may reduce the cardiac event rate (76).

All patients with confirmed cardiotoxicity require referral to a cardio-oncology service. Any patient with pre-existing LV systolic dysfunction should be discussed with the cardio-oncology team, ideally before the commencement of cardiotoxic cancer therapy (2). With increasing evidence that a significant reduction in GLS accurately predicts subsequent cardiotoxicity (35), referral to a cardio-oncology service for expert review could be considered in patients with > 15% reduction in GLS despite a normal LVEF.

## Treatment thresholds

Patients who develop CTRCD (symptomatic or asymptomatic) benefit from early introduction of angiotensin-converting enzyme inhibitors/angiotensin receptor blockers and/or beta-blockers (77). Treatment thresholds vary in the published reports (10, 78, 79). Treatment in asymptomatic patients with declines in GLS, but not in LVEF, remains controversial (13, 80). Closer monitoring and/or starting cardioprotective treatments are options to be considered. There is no evidence at present to hold cancer therapy based upon abnormal strain measurements alone.

https://erp.bioscientifica.com
https://doi.org/10.1530/ERP-21-0001
© 2021 The authors
Published by Bioscientifica Ltd
This work is licensed under a Creative Commons Attribution-NonCommercial-NoDerivatives 4.0 International License.

Recent studies have investigated the role of pre-treatment with cardioprotective medications in cancer patients embarking on cardiotoxic therapy (81, 82, 83). Results have been mixed, and such strategies cannot currently be recommended as routine practice.

## Conclusions

Cardio-oncology is a relatively new and rapidly developing subspecialty. Echocardiography is a key imaging modality in the initial assessment and subsequent monitoring of patients treated with commonly used cardiotoxic drugs such as anthracyclines and trastuzumab. High-quality targeted LVEF assessment incorporating 3D volumetric analysis and strain measurement can be used to safely monitor patients during and after treatment. Key echocardiographic recommendations are shown in Table 5.

### Declaration of interest

The authors declare that there is no conflict of interest that could be perceived as prejudicing the impartiality of this guideline.

### Funding

This work was supported by National Institutes of Health grant R01 HL 118018 (Dr Ky).

### Author contribution statement

R Dobson and A K Ghosh contributed equally to this work. Anju Nohria, MD, served as the Editor-in-Chief for this paper. Juan Carlos Plana Gomez, MD, served as the Guest Associate Editor for this paper.

### Acknowledgements

Anju Nohria, MD, served as the Editor-in-Chief for this paper. Juan Carlos Plana Gomez, MD, served as the Guest Associate Editor for this paper.
